# Taxonomic and morphological survey of the *Lygephila lusoria* (Linnaeus, 1758) species-group with description of a new species (Lepidoptera, Erebidae, Toxocampinae)

**DOI:** 10.3897/zookeys.351.5999

**Published:** 2013-11-15

**Authors:** Oleg Pekarsky

**Affiliations:** 1 H-1068, Budapest, Felsőerdősor u. 16-18, Hungary

**Keywords:** Lepidoptera, Erebidae, Toxocampinae, *Lygephila lusoria* species-group, new species, vesica structure, Russia, Europe

## Abstract

The taxa of the *Lygephila lusoria* (Linnaeus, 1758) species-group are revised. The genital features of all known taxa are described and illustrated with special reference to the structure of vesica. The male genitalia of *L. pallida pallida* (Bang-Haas, 1907) are described and illustrated for the first time. *L. pallida subpicata* (Wiltshire, 1971) is treated here as a species, *L. pallida subpicata* (Wiltshire, 1971), **stat. n.**, distinct from *L. pallida*. A new species, *L. minima*, **sp. n.**,from South Russia is described. Illustrations of the holotype and its genitalia are provided; a diagnostic comparison with *L. pallida* is given. *L. alaica* Remm, 1983 is included in the *L. lusoria* species-group for the first time.

## Introduction

The genus *Lygephila* Billberg, 1820 is a popular group; several authors have published new results concerning the taxonomy, faunistics and bionomics of the group during the last century, increasing our knowledge of this diverse and taxonomically difficult group. The recent wave of investigations, after the works of Bryk (1948), Draudt (1950), Sheljuzhko (1955), Wiltshire (1961, 1971), Sugi (1982), Remm (1983), Behounek and Hacker (1986), Kinoshita and Sasaki (1986), Kinoshita (1989), Calle (1983), Sviridov (1990), Yela and Calle (1990), Yela (1989), produced remarkable results published by Kononenko and Fibiger (2008), Fibiger et al. (2008), Bertaccini et al. (2008), Babics and Ronkay (2009) and Babics and Stüning (2011). The sketch of the species content of the genus and the characterisation of certain species-groups are published by Goater et al. (2003) and Babics and Ronkay (op. cit.). Following this wave of publications, I intend to revise the species-groups of *Lygephila*, concentrating the study on the formerly neglected types and structure of the vesica. The present paper is addressed to the taxonomy of the *Lygephila lusoria* species-group.

**Abbreviations of material depositories** BMNH = The Natural History Museum (British Museum, Natural History) London (United Kingdom), HNHM = Hungarian Natural History Museum Budapest (Hungary); MA= Matov Alexey, St. Petersburg (Russia); MNHN = Museum National d’Histoire Naturelle Paris (France); MNHU = Museum für Naturkunde der Humboldt-Universität zu Berlin (Germany); NHMW = Naturhistorisches Museum Wien (Vienna, Austria); ZISP = Zoological Institute, Russian Academy of Sciences St. Petersburg (Russia); OP = Oleg Pekarsky, Budapest (Hungary).

## Materials and methods

Male and female genitalia were dissected and mounted in euparal on glass sides. Photos of genitalia where made by Svitlana Pekarska using microscope Nikon SMZ745T and camera Moticam 2500. Photos of imago where taken by the author using camera Nikon D3000/Sigma 105, f/2.8.

## Systematic accounts

### Description of the *Lygephila lusoria* species-group

Head and body greyish brown with frons and collar chocolate brown. Forewing in general broad, elongated with pointed apex, greyish brown with indistinct transverse lines, orbicular stigma dot-like, reniform stigma more or less triangular, black, sometimes with sharp extension at inner corner and satellite streak-like spots on outer margin; hindwing with wide outer band and visible discal spot. The first characterisation of the genitalia structure of the *Lygephila lusoria* species-group was given by Babics and Ronkay in 2009 and this characterisation is to be revised and amended. Some of the previously mentioned characters, such as the strong, sabre-shaped uncus being broadened in third quarter, and having an acute tip, the elongated valva with more or less parallel margins, the wide, funnel-shaped ostium bursae, and the membranous, elliptical corpus bursae are shared features with the *Lygephila lubrica* species-group. Therefore, these characters cannot be considered as distinctive features for the *Lygephila lusoria* group. The autapomorphies of this group can be found in the shape of the ampulla, the aedeagus, the vesica structure and some of the specific parameters of the ostium bursae. The ampulla is tapered with a long, skewed base, which is comparable in length in practically all members of the group. The aedeagus is short and wide; the vesica has six or seven diverticula, the subbasal diverticulum is well developed with a ridge-like cornutus complex. The female genitalia are characterised by the markedly asymmetrical ostium bursae, in comparison with the other species groups of the genus.

The *Lygephila lusoria* species-group comprises the following species: *Lygephila lusoria lusoria* (Linnaeus, 1758); *Lygephila lusoria glycyrrhizae* (Rambur, 1866); *Lygephila amasina* (Staudinger, 1878); *Lygephila colorata* Babics & Ronkay, 2009; *Lygephila moellendorffi* (Herz, 1904); *Lygephila pallida* (Bang-Haas, 1907); *Lygephila subpicata* Wiltshire, 1971, stat. n.; *Lygephila minima* sp. n.; *Lygephila fereidun* Wiltshire, 1961; *Lygephila alaica* Remm, 1983.

#### Lygephila lusoria lusoria

(Linnaeus, 1758)

http://species-id.net/wiki/Lygephila_lusoria_lusoria

Figs 1–4

##### Material examined.

1 ♂, Hungary, Pilisszántó, Üdülőtelep, Plachkó u., 18.VI.2007, leg. & coll. O. Pekarsky slide No: OP1953m, 1 ♀, Hungary, Naszály, Sejce, N47°50'304, E019°06'762, 11.VI.2007 leg. & coll. O. Pekarsky, slide No: OP1954f; 1 ♂, 1 ♀, Süd-Frankreich, Provence Serres, 4 km südlich Orpierre, 1000 m, 18.07.1999, leg. P. Kuhna, coll. ZFMK, slide Nos: OP2263m, OP2264f; 1 ♂, 1 ♀, Crimea, Alushta, Luchistoe, South Demergi Mt., 16.06.2012, leg. V. Savchuk, coll. N. Kaygorodova, slide Nos: OP2052m, OP2053f; 1 ♂, Russia, S Ural, Orenburg Obl., Kuvandyk, 23–24.6.2009, leg. & coll. L. Srnka, slide No: OP2122m.

##### Diagnosis.

*Lygephila lusoria lusoria* is the largest representative of the species group. Differ from *Lygephila amasina* by less contrast wing pattern and not sharp inner corner of the reniform stigmata. Nominotypical subspecies in most cases lager, with more contrast wing pattern comparing with *Lygephila lusoria glycyrrhizae* from Spain.

**Male genitalia** (Figs 25, 39, 40). Uncus stem narrow and relatively long, dilated distally with fine tip, scaphium membranous with sclerotized plate on subscaphium; valva elongated, narrowed at base, apex rather acute; ampulla spine-like, almost straight, not reaching apex of valva, its base asymmetrical. Aedeagus straight, tubular, slightly dilated at carina with heavily sclerotized field on it. Vesica globular, everted forward and recurved laterally; medial part membranous; subbasal diverticulum oblate with heavily sclerotized crest contacting carina; 1^st^ medial diverticulum small; 2^nd^ and 3^rd^ medial diverticula elongated, tube-like, rising from extension of main vesica chamber located opposite to each other; 4^th^ medial diverticulum on opposite side topped with large, rounded, plate-like cornutus with two teeth; 1^st^ terminal diverticulum tapered with large basal swelling; 2^nd^ terminal diverticulum tapered, bordering 2^nd^ medial diverticula, bearing three small pockets; terminal tube membranous with weak scobination at end near gonopore (starting point of ductus ejaculatorius), opening point of terminal tube located at base of medial part of vesica near carina. **Female genitalia** (Figs 76, 77). Ovipositor relatively short, broad; papillae anales hairy with long setae on apical edges. Apophyses anteriores slender, apophyses posteriores thin with acute tips, longer than apophyses anteriores. Antrum tapering, ostium bursae broad with acute lateral edges, posterior margin incised producing shallow triangular cleft with almost straight margins; ductus bursae large, wide with coarse well-sclerotized wrinkles laterally. Appendix bursae small with ductus seminalis located near ductus bursae. Corpus bursae membranous, large, ellipsoidal.

##### Distribution.

West Palearctic. In Europe it ranges from Spain to Bulgaria, from Ukraine to south Russia and western Kazakhstan (Uralsk). All earlier records for Asia Minor refer to *Lygephila amasina*, whereas the records from north Caucasus and Transcaucasia belong to *Lygephila minima* sp. n.

**Figures 1–8.**
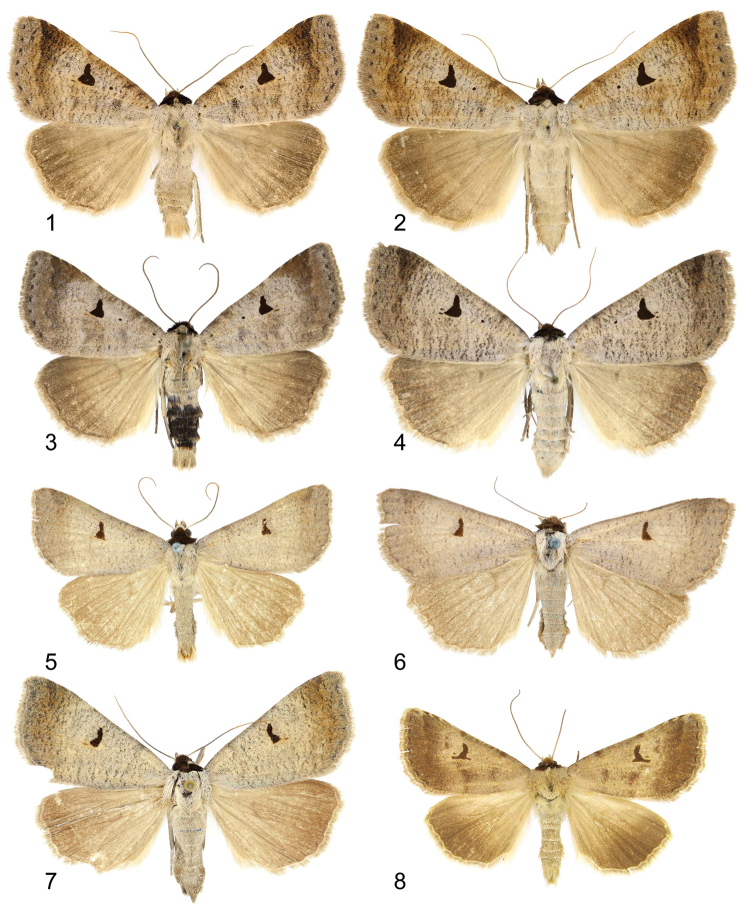
Adults. **1–4** *Lygephila lusoria lusoria* **1** male, Hungary, slide No. OP1953m **2** female, Hungary, slide No. OP1954f **3** male, S Ural, slide No. OP2122m **4** female, France, slide No. OP2264f **5–7** *Lygephila lusoria glycyrrhizae* **5** male, Spain, Andalusia, slide No. OP1977m **6** female, Spain, Andalusia, slide No. OP1978f **7** female, Spain, Granada, slide No. OP2265f **8** *Lygephila moellendorffi* paratype, male, N Korea (photo A. Matov).

#### Lygephila lusoria glycyrrhizae

(Rambur, 1866)

http://species-id.net/wiki/Lygephila_lusoria_glycyrrhizae

Figs 5–7

##### Material examined.

1 ♂, Andalusien, Sierra de Alfacar, 1905, C. Ribbe, coll. MNHU Berlin, slide No: OP1977m; 1 ♀, Andalusien, Sierra de Alfacar, 1905, C. Ribbe, coll. MNHU Berlin, slide No.: OP1978f; 1 ♀, Espania, S. Albarracin, 1500m, (Teruel), 8.7.1987, leg. Fidel Fernandez-Rubio, coll. P. Gyulai, slide No.: OP2128f; 1 ♀, Spain, Sierra de Bata, Sta Barbara, Granada, 1800 m, 30.vi.1994, leg. B. Goater, coll. G. Ronkay, slide No.: OP2137f; 1 ♀, Spanien, Granada, Sierra Nevada, Pico Valeta, 2500 m, 4.7.1987, leg. P. Kuhna, coll. ZFMK, slide No.: OP2137f.

##### Note.

The name of this taxon is unavailable from Rambur, 1866, and there is some debate as to the correct authorship and date of this subspecies. This issue will be dealt with in a separate publication.

##### Diagnosis.

This taxon was downgraded to a subspecies of *Lygephila lusoria* by Bertaccini et al. (2008). It is interesting that, despite the remarkable external differences between *Lygephila lusoria lusoria* and *Lygephila lusoria glycyrrhizae*,no valuable differences can be recognised in the male and female genitalia of the two taxa. The most significant distinctive feature of *Lygephila lusoria glycyrrhizae* is, in comparison with *Lygephila lusoria lusoria*, the small size of the genitalia of both sexes. The genitalia of the Spanish moths are approximately 1.3 times smaller than those of *Lygephila lusoria lusoria* from Central and Eastern Europe, Crimea and Urals. In addition, there are a few hardly recognisable differences in the shape of uncus, valva and aedeagus: *Lygephila lusoria glycyrrhizae* has somewhat shorter uncus stem and valvae with costal dilatation medially and less curved aedeagus, whereas the plan of the female genitalia of the two taxa is practically the same.

**Male genitalia** (Figs 26, 41, 42). Uncus stem narrow and relatively short, dilated distally with fine tip; scaphium membranous with sclerotized plate on subscaphium; valva elongated, narrowed at base, margins not parallel due to large costal dilatation medially, valval apex rather acute; ampulla almost straight, spine-like with symmetrical base. Aedeagus a straight tube with heavily sclerotized field on carina. Vesica globular, everted forward and recurved laterally; medial part membranous; subbasal diverticulum oblate with heavily sclerotized crest contacting carina; 1^st^ medial diverticulum small; 2^nd^ and 3^rd^ medial diverticula elongated, tube-like, rising from extension of main vesica chamber, located opposite to each other; 4^th^ medial diverticulum on opposite side topped with large, rounded, plate-like cornutus with two teeth; 1^st^ terminal diverticulum tapered, with large basal swelling; 2^nd^ terminal diverticulum bears three small pockets; terminal tube membranous with weak scobination at distal end near gonopore (starting point of ductus ejaculatorius); opening point of terminal tube located at base of medial part of vesica near to carina. **Female genitalia** (Figs 78–80). Ovipositor relatively short, broad, papillae anales hairy with long setae on apical edges. Apophyses anteriores slender, apophyses posteriores longer than apophyses anteriores, thin with acute tips. Antrum tapering, ostium bursae broad with acute lateral edges, posterior margin incised showing shallow triangular cleft with almost straight margins; ductus bursae large, wide with coarse well-sclerotized wrinkles laterally. Appendix bursae small with ductus seminalis located near ductus bursae. Corpus bursae membranous, large, elongated, ellipsoidal.

##### Distribution.

Spain.

#### Lygephila amasina

(Staudinger, 1878)

http://species-id.net/wiki/Lygephila_amasina

Figs 9 , 10

##### Material examined.

1 ♂, Turkey, Prov. Agri, Karasu-Aras Mts, 2100m, 7km E from Aydintepe, 42°28'27"E, 39°47'4"N, 04.VII.2000, leg. Gy. Fábián, I. Szécsényi & K. Székely, coll. O. Pekarsky, slide No.: OP1959m; 1 ♂, Türkei, 12 km west Ürgüp, 1400 m, 21.6.1979, leg. P. Kuhna, coll. ZFMK, slide No. OP2260m; 1 ♀, Türkei, 12 km west Ürgüp, 1400 m, 11.9.1981, leg. P. Kuhna, coll. ZFMK, slide No. OP2261f; 1 ♀, Libanon, Jabal el Laqlouq, Street Mkhada–Laqloq, 1300–1500 m NN, 13.06.1999, leg. J. Krüger, coll. ZFMK, slide No. OP2262f; 1 ♀, Lebanon, Laqlouq, h-1600m, 25.07.2011, leg. Floriani & Saldaitis, coll. O. Pekarsky, slide No.: OP1960f.

##### Diagnosis.

*Lygephila amasina* distinguishing from similar *Lygephila lusoria lusoria* by more contrast wing pattern and somewhat longer, sometimes with acute apex of inner corner of the reniform stigmata. In genital structures it differs from *Lygephila lusoria* by broader uncus, longer, thinner ampulla reaching apex of valva, not sharp lateral edges of antrum and ovoid corpus bursa.

**Male genitalia** (Figs 27, 43–46). Slightly asymmetrical (right valva narrower). Uncus short, dilated distally, with fine tip; scaphium membranous with sclerotized plate on subscaphium; valva elongated, narrowed at base with apex rather acute; ampulla long, stick-like, slightly curved towards costa, reaching apex of valva. Aedeagus short with heavily sclerotized convex field on carina and spinulose area on lamina. Vesica small, globular, everted laterally; medial part membranous; heavily sclerotized crest with ridge-like cornutus complex based on elongated oblate diverticulum-like subbasal hump; 1^st^ medial diverticulum medium-sized, 2^nd^ medial diverticulum much larger, located on the opposite side of vesica with sclerotized area on the top; 1^st^ terminal diverticulum two-chambered, one of them elongated tapering, another globular; 2^nd^ terminal diverticulum tapering with acute top; 3^rd^ terminal diverticulum situated in the same line with 2^nd^ medial diverticulum; opening point of terminal tube located at base of medial part of vesica near to carina; terminal tube membranous with narrow sclerotized crest at base and weak scobination at distal end near gonopore (starting point of ductus ejaculatorius). **Female genitalia** (Fig. 81). Ovipositor short, papillae anales hairy with long setae on apical edges. Apophyses anteriores slender, apophyses posteriores thin, 1.6 times longer than apophyses anteriores. Antrum infundibuliform, asymmetrical, with heavily sclerotized elongated plate dorsally; ostium bursae broad, posterior margin gently concave; ductus bursae practically absent. Appendix bursae indistinct. Corpus bursae membranous, ovoid.

##### Distribution.

Turkey, Lebanon and Israel.

**Figures 9–16.**
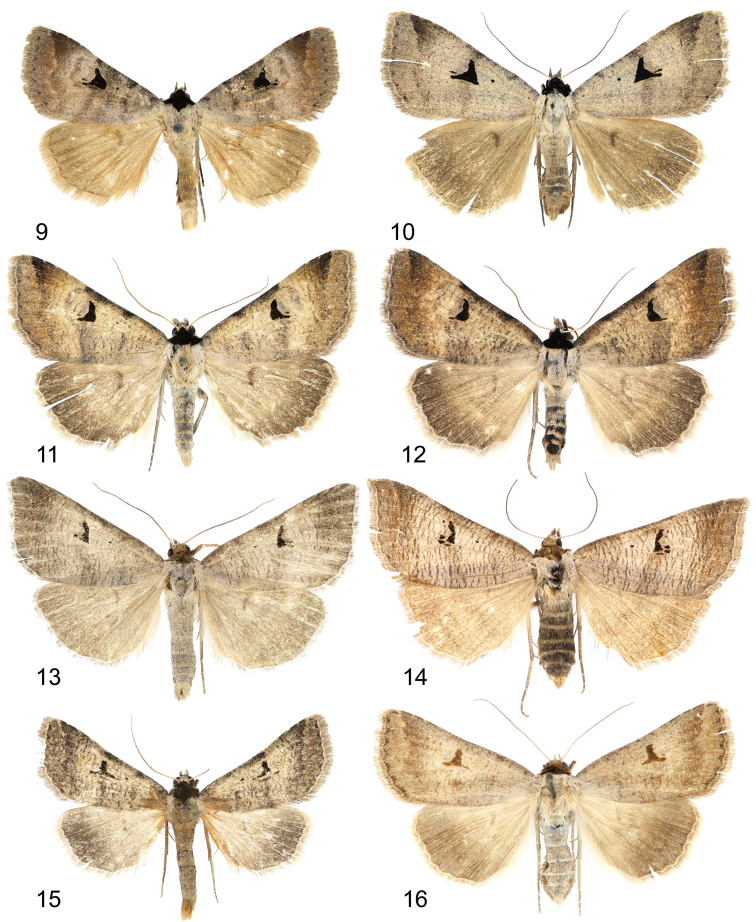
Adults. **9, 10** *Lygephila amasina* **9** male, Turkey, slide No. OP1959m **10** female, Lebanon, slide No. OP1960f **11, 12** *Lygephila colorata* **11** paratype, male, Pakistan, slide No. OP1969m **12** paratype, female, Pakistan, slide No. OP1970f **13, 14** *Lygephila alaica* **13** male, Tajikistan, slide No. OP1819m **14** female, Uzbekistan, slide No. OP1792f **15, 16** *Lygephila subpicata* **15** male, Iran, Zagros Mts, slide No. OP2002m **16** paratype, female, Iran, Semnan slide No. OP2060f.

#### Lygephila colorata

Babics & Ronkay, 2009

http://species-id.net/wiki/Lygephila_colorata

Figs 11 , 12

##### Material examined.

1 ♂, Paratype, Pakistan, Karakoram Mts, Naltar valley, 2800m, 74°12'E, 36°09.6'N, 30.06.2000, leg. Z. Varga & G. Ronkay, coll. O. Pekarsky, slide No.: OP1969m; 1 ♀, same data as male, slide No.: OP1970f.

##### Diagnosis.

*Lygephila colorata* differ from somewhat externally similar *Lygephila amasina* by more elongated forewing with pointed apex. In male genitalia it differ from congeners by very wide, massive valva, strong, thick ampulla. Female genitalia characterised by deeply concave posterior margin of antrum.

**Male genitalia** (Figs 30, 47–51). Clasping apparatus slightly asymmetrical (right valva narrower). Uncus short, dilated medially, apex with fine tip; scaphium membranous with sclerotized plate on subscaphium; valva elongated, narrowed at base with rather rounded apex (right valva more acute); ampulla large, massive, slightly curved towards costa, with obtuse tip. Aedeagus short, bent medially, with heavily sclerotized convex field on carina and spinulose area on lamina. Vesica large rather globular, multidiverticulate, everted laterally; medial part membranous; 1^st^ subbasal diverticulum bearing heavily sclerotized crest with ridge-like cornutus complex; 2^nd^ subbasal diverticulum bifurcated, composed from two narrow, elongated tube-like diverticula; medial diverticulum large, elongated, S-shaped with bilobate base and tapering upper part with acute tip; large, elongated terminal complex consists of five diverticula, one of them with densely scobinated top; opening point of terminal tube located at base of vesica near the carina; terminal tube membranous with slightly sclerotized area at base and weak scobination near gonopore (starting point of ductus ejaculatorius). **Female genitalia** (Fig. 82). Ovipositor short, papillae anales small, hairy with long setae on apical edges. Apophyses anteriores slender with fine tip, apophyses posteriores thin, somewhat longer than apophyses anteriores. Antrum U-shaped, asymmetrical, ostium bursae broad, posterior margin deeply concave; ductus bursae small. Appendix bursae small. Corpus bursae membranous, ovoid.

##### Distribution.

North-western Pakistan.

#### Lygephila pallida

(Bang-Haas, 1907)

http://species-id.net/wiki/Lygephila_pallida

Figs 17 , 19 , 20

##### Material examined.

1 ♂, Cotype label1: Cotype, pallida B.-H. ♂; label2: As. min. m. (Zeitun), revers - pallida B.-H. ♂, 5/08 vom Autor; label3: Zeitun; label4: 962; ex. coll. Püngeler, coll. MNHU Berlin, slide No: OP1933m; 1 ♂, Turkey, Prov. Kayseri, 5 km NW Ercios Dagh, 2000 m, 22.7.1986, leg. M. Fibiger, coll. G. Ronkay, slide No: OP1967m; 1 ♂, Turkey, Prov. Sivas, Ziyaret gecidi, 2100 m, 36°45'E, 38°42'N, 27–28.07.1993, leg. Gy. László, coll. O. Pekarsky, slide No: OP1961m; 1 ♀, Turkey, Prov. Sivas, Ziyaret gecidi, 1950–2050 m, 36°45'E, 38°42'N, 27.07.1988, leg. Gyulai, Hreblay, Ronkay & Ronkay, coll. G. Ronkay, slide No: OP1966f; 1 ♀, Türkey, Prov. Sivas, 5 km E of Imranli, 38°06'E, 39°53'N, 11.VII.1989, leg. & coll. P. Gyulai, slide No: OP2014f; 1 ♂, [Turkey] O Anatolien, Gürün, 4.VII.76, leg. Pinker, coll. NHMW, Vienna, slide No: OP2065m; 1 ♀, [Turkey] O Anatolien, Gürün, 4.VII.76, leg. Pinker, coll. NHMW, Vienna, slide No: OP2066f; 1 ♂, Turkey, Prov. Erzurum-Erzincan, 10 km W of Askale, 1700m, 40°34'E, 39°50'N, 08.08.1988, leg. Gyulai, Hreblay, Ronkay & Ronkay, coll. G. Ronkay, slide No: OP2029m; 1 ♂, Turkey, Prov. Erzurum, 4 km W of Tahir, 2500 m, 42°27'E, 39°51'5"N, 22.07.1993, leg. Gy. László, coll. G. Ronkay, slide No: OP2030m; 1 ♂, Türkei, Palandoeken, 2500 m, 28 Juli 1980, leg. Dittrich Austria, coll. NHMW, Vienna, slide No: OP2069m; 1 ♀, Türkei, Palandoeken, 2500 m, 28 Juli 1980, leg. Dittrich Austria, coll. NHMW, slide No.: OP2070f; 1 ♀, Turkey, Prov. Agri, 7 km W of Aydintepe, 2200 m, 42°30'E, 39°49'N, 20–22.VII.1990, leg. Gy. László & G. Ronkay, coll. G. Ronkay, slide No: OP1968f.

##### Diagnosis.

Distinguishable from similar species only by genitalia characters. It differ from *Lygephila subpicata* by shorter spine-like ampulla not reaching the valval edges and from *Lygephila minima* sp. n. by narrower valva, longer ampulla and absents of sclerotization on top of the 2^nd^ medial diverticulum.

**Male genitalia** (Figs 31, 52–59). Clasping apparatus somewhat asymmetrical (right valva narrower). Uncus stem narrow, short, dilated distally, with fine tip; scaphium membranous with weakly sclerotized plate on subscaphium; valva elongated, narrowed at base with rather acute apex; ampulla spine-like, slightly curved towards costa, finely pointed, does not reaching apex of valva. Aedeagus short, slightly curved medially, with heavily sclerotized convex field on carina and spinulose area on lamina. Vesica globular, everted forward and recurved laterally; medial part membranous; heavily sclerotized ridge on subbasal diverticulum with cornutus complex contacting carina at base; 1^st^ medial diverticulum medium-sized, wide at base; 2^nd^ medial diverticulum very large, conical, with sclerotized area on the top; 1^st^ terminal diverticulum two chambered, one of them elongated tapering, another globular; 2^nd^ terminal diverticulum tapered; 3^rd^ terminal diverticulum elongated with very wide base and curved tapered part; opening point of terminal tube located at base of medial part of vesica near carina, terminal tube membranous with narrow sclerotized crest at base and weak scobination near gonopore (starting point of ductus ejaculatorius). **Female genitalia** (Fig. 83). There were no females with type labels or from the same collecting place as the cotype in the MNHU collection. Taking into consideration that the exemplar from Palandöken, Turkey is the most similar in male genitalia structure to the cotype specimen (the two slides are almost fully agree with each other) one can conclude that the female specimen from the same site would represent the female sex of *Lygephila pallida*. Ovipositor short, papillae anales small, hairy with long setae on apical edges. Apophyses anteriores slender with fine tip, apophyses posteriores thin, somewhat longer than apophyses anteriores. Antrum U-shaped, asymmetrical, ostium bursae broad, posterior margin deeply concave with large prolongation of posterior end on one side; ductus bursae small, practically absent. Appendix bursae small. Corpus bursae membranous, ovoid.

##### Distribution.

Central and eastern Turkey.

**Figures 17–24.**
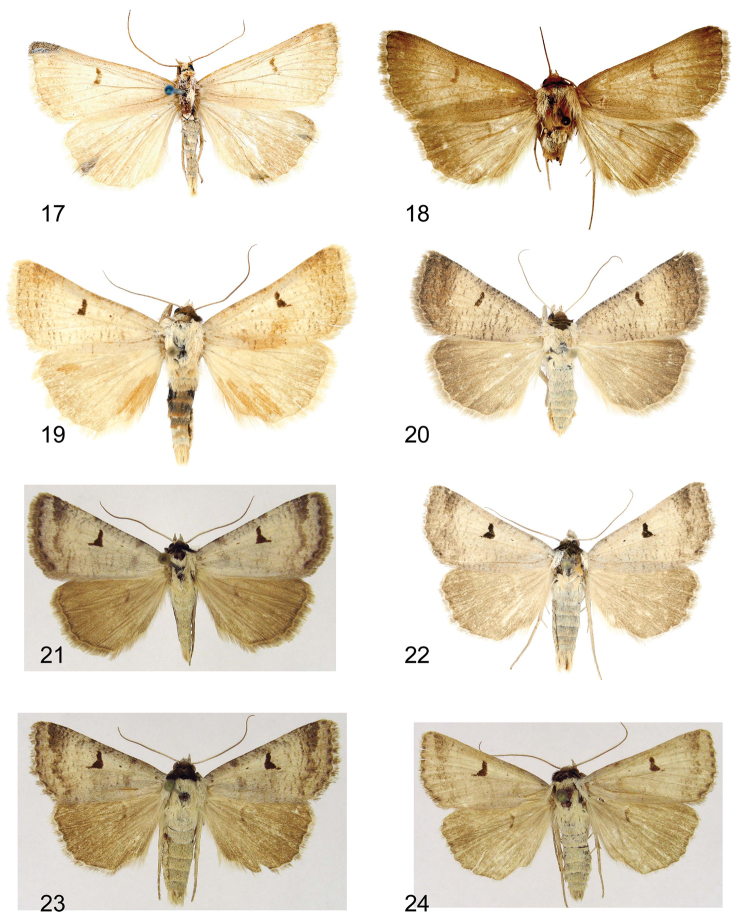
Adults. **17, 19, 20** *Lygephila pallida* **17** Cotype, male, Turkey, Zeitun, slide No. OP1933m **18** *Lygephila fereidun* holotype, male, Iran, Elburz (photo G. Ronkay) **19** male, Turkey, Prov. Sivas, slide No. OP1961m **20** female, Turkey, Prov. Sivas, slide No. OP2014f **21–24** *Lygephila minima* sp. n. **21** holotype, South Russia, Stavropol krai, slide No. 0329Matov (photo A. Matov) **22** paratype, male, South Russia, Stavropol krai, slide No. OP1607m **23, 24** paratypes, males, South Russia, Stavropol krai (photo A. Matov).

**Figures 25–30.**
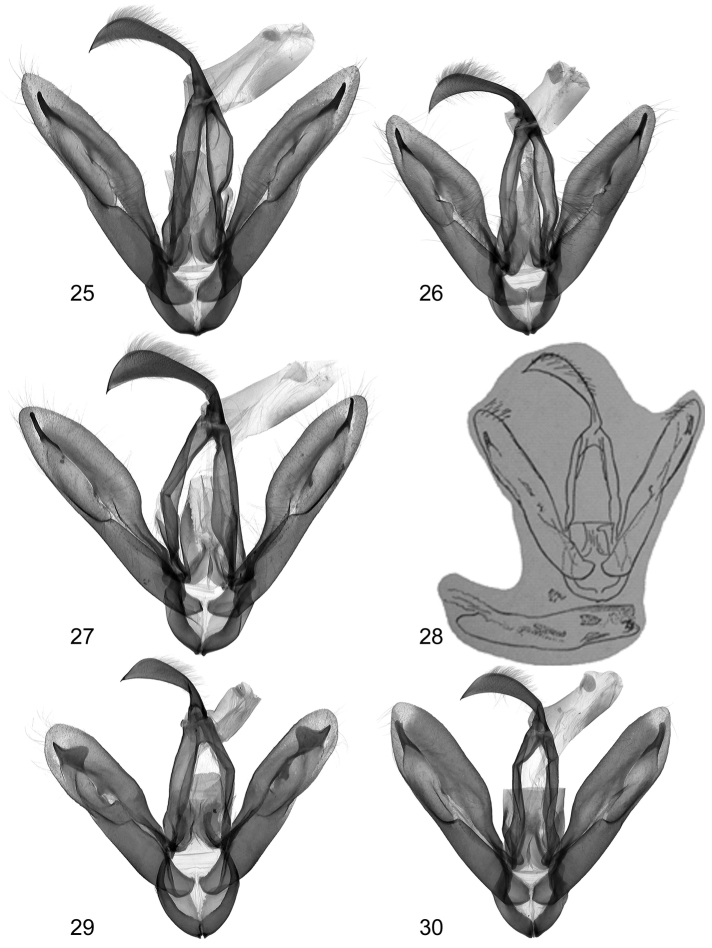
Clasping apparatus **25** *Lygephila lusoria lusoria* Hungary, slide No. OP1953m **26** *Lygephila lusoria glycyrrhizae* Spain, slide No. OP1977m **27** *Lygephila amasina* Turkey, slide No. OP1959m **28** *Lygephila fereudun* Type, Iran, Elburz, after Wiltshire (1961) **29** *Lygephila alaica* Tajikistan, Gissar Mts, slide No. OP1819m **30** *Lygephila colorata* paratype, Pakistan, slide No. OP1969m.

**Figures 31–38.**
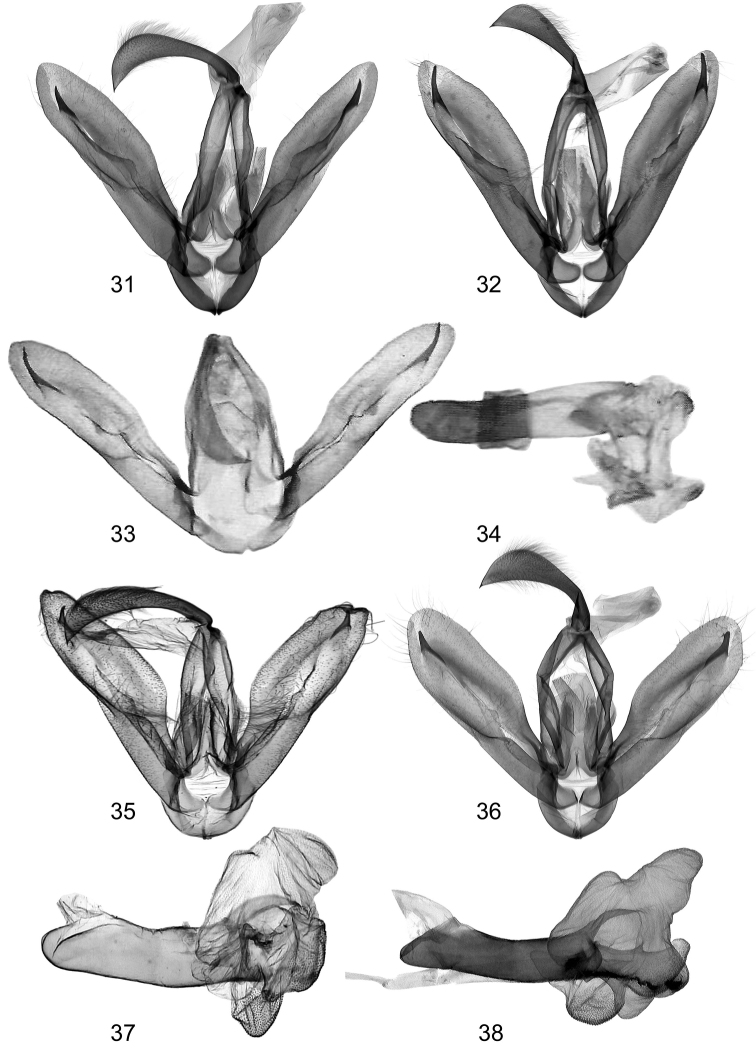
Clasping apparatus **31** *Lygephila pallida* Cotype, Turkey, Zeitun, slide No. OP1933m **32** *Lygephila subpicata* Iran, Prov. Fars, slide No. OP2002m **33, 34** *Lygephila moellendorffi* paralectrotype, N Korea, slide No. VK210394-10 ZIN (photo V. Kononenko) **35, 37** *Lygephila minima* sp. n. holotype, South Russia, Stavropol krai, slide No. 0329Matov (photo A. Matov) **36, 38** paratype, male, South Russia, Stavropol krai, slide No. OP1607m.

#### Lygephila fereidun

Wiltshire, 1961

http://species-id.net/wiki/Lygephila_fereidun

Fig. 18

##### Taxonomy.

This taxon, described from the Elburz Mountains, Northern Iran, is known only from the holotype (coll. BMNH). In the original description the color was characterized as pale straw and the wing pattern close to the Spanish species *glycyrrhizae*. The diagnostic comparison was made with *Lygephila craccae* ([Denis & Schiffermüller], 1775) and *Lygephila lusoria* only, whereas a comparison with another similar species, *Lygephila pallida*,was neglected. The original description contains the following text about the clasping apparatus structure (Fig. 28): “The harpe [ampulla], longer than that of *craccae*, is nevertheless shorter than that of *lusoria.*” Comparative analysis of the ampullar length (shorter than that of *lusoria*) given by Wiltshire, makes it possible to conclude that the *Lygephila fereidun* is different from the *Lygephila amasina* and *Lygephila subpicata*, because they have longer ampullae that reach the costal margin of the valva. So, by this feature *Lygephila fereidun* could be compared only with *Lygephila pallida*,the ampulla of which is rather shorter than that of *Lygephila lusoria* and other members of its species group. Vesica structure in the original description is characterized as follows: “The vesica contains similar elements to those of *lusoria* but the proximal scobinated field is shorter and the five or six teeth on the distal chitinous lump are larger and more like cornuti than in *lusoria*.” However, the only sclerotized cornutus formation illustrated in the original drawing looks similar to that of *Lygephila subpicata*, but *Lygephila subpicata* has two heavily-sclerotized crown-like cornuti on the top of subbasal and 2^nd^ medial diverticula.

The above-mentioned contradictions in the original description thereby make it impossible to clarify the taxonomical situation of this taxon without a study of the genitalia of the holotype, the preparation of which is opaque and requires specific recovery treatment. Based on the currently known characters *Lygephila fereidun* is most likely an aberrant specimenof *Lygephila pallida*.

##### Distribution.

Northern Iran.

#### Lygephila minima sp. n.

http://zoobank.org/ED5224B3-3A40-4A84-8EFA-F4F498213211

http://species-id.net/wiki/Lygephila_minima

Figs 21–24

##### Type material.

**Holotype:** Male (Fig. 21), [Russia], Stavropolskiy krai, NW suburbs of station Podkumok, 26.06.2008, leg. E. Tsvetkov, slide No.: 0329Matov (coll. ZISP)

**Paratypes.** Males. 1 ♂, [Russia], Stavropolskiy krai, suburbs of Pyatigorsk, station Podkumok, 20.07.2007, leg. E. Tsvetkov; slide No.: OP1607m (coll. O. Pekarsky). 2 ♂♂, [Russia], Stavropolskiy krai, suburbs of station Podkumok, 43°57'43''N, 42°46'18''E, leg. E. Tsvetkov, 20.07.2007; 1 ♂, [Russia], Stavropolskiy krai, suburbs of Piatigorsk, station Podkumok, 18.07.2007, leg. E. Tsvetkov; 1 ♂, Armenia, Daralagez, 12.VIII.[19]63, slide No.: 0341Matov (coll. ZISP).

##### Etymology.

The name “*minima*” refers to the small size of the moth in contrast to the largest representative of the genus, *Lygephila maxima* (Bremer, 1861).

##### Diagnosis.

The new speciesresembles *Lygephila pallida* by its small size and pale brown ground color of the forewing. *Lygephila minima* differs from the related species by its better developed noctuid pattern, more rounded wings and pale grey-brown ground color of the forewings. Apical dilatation of uncus wide, valva wide with rounded apex, ampullar tip not sharp, 1^st^ medial diverticulum reniform; 2^nd^ medial diverticulum hemispherical, membranous, without sclerotized areas, whereas *Lygephila pallida* has narrower dilatation at the top of the uncus, longer, narrower valva with acute apex, fine tipped ampulla, 1^st^ medial diverticulum very wide at base, swelling-like; 2^nd^ medial diverticulum large, tubular, with sclerotized area on the top.

##### Description.

Male (Figs 21–24). Wingspan 33 mm, length of forewing 17 mm. Head and collar coffee brown. Palpi short, relatively narrow, beige; antenna filiform. Thorax and abdomen beige. Forewing beige with silver shining, irrorated with a few blackish-brown scales; forewing short, wide; costa straight; outer margin rounded; wing pattern indistinct: basal, subbasal and antemedial lines hardly recognisable; medial line represented by large costal patch and some darker spots medially; postmedial line indistinct; subterminal line curved, composed by blackish-brown scales; terminal line marked by large triangular patches; cilia long, uniformly light brown; orbicular stigma dot-like, as coffee-brown colored as V-shaped reniform; claviform stigma indistinct. Hindwing beige brown, discal spot narrow. Female unknown.

**Male genitalia** (Figs 35–38, 60–67). Uncus stem short, broadly dilated distally with fine tip; valva short, wide, rounded apically with rather parallel margins in distal two-thirds, slightly narrower at base; ampulla spine-like with long base and pointed tip which does not reaching margin of valva. Aedeagus short, curved medially, with heavily sclerotized field on carina and spinulose area on lamina. Vesica globular, everted forward and recurved laterally; medial part membranous; basal cornutus ridge interrupted without sclerotized base, subbasal diverticulum medium sized; 1^st^ medial diverticulum large, reniform; 2^nd^ medial diverticulum hemispherical; 3^rd^ medial diverticulum tapered, 1^st^ distal diverticulum large, subconical, 2^nd^ distal diverticulum with wide base and crooked tip; opening point of terminal tube located at base of medial part of vesica, terminal tube membranous with sclerotized ribbon at base and weak scobination at end near gonopore.

##### Distribution.

The species is known from south Russia, Stavropol region and Armenia.

#### Lygephila subpicata

(Wiltshire, 1971) stat. n.

Figs 15 , 16

##### Material examined.

1 ♂, 1 ♀ Paratypes, N-Iran, Berge östl. Semnan, 18.VI.1963, leg. Kasy & Vartian, coll. NHMW, slide Nos: OP2059m, OP2060f; 1 ♂, 1 ♀ S-Iran, 100 km südl. Abadeh, nördl Didegan, 2000 m, 9.6.1969, leg. Vartian, coll. NHMW, slide Nos: OP2061m, OP2062f; 2 ♂♂ Iran, Prov. Fars, Zagros Mts, Ardakan, 2500–3000 m, 18.VI.2010, leg. B. Benedek & T. Hácz, coll. P. Gyulai, slide Nos: OP2002m, OP2003m.

##### Diagnosis.

*Lygephila subpicata* differs from its sister species, *Lygephila pallida* in the length and shape of the ampulla, and in vesica and aedeagus structure. *Lygephila subpicata* has a much longer, curved ampulla, which reaches apex of valva and costal margin; subbasal diverticulum large with crown-like cornutus on top, tapering part of 1^st^ terminal diverticulum small, short and narrow, 2^nd^ medial diverticulum with crown-like cornutus on top, carinal extension practically absent. In comparison, *Lygephila pallida* has shorter, less curved, finely pointed ampulla that does not reach apex of valva, a small, oblate subbasal diverticulum with a long, heavily-sclerotized, ridge-like cornutus complex that is a continuation of the carina.

**Male genitalia** (Figs 32, 68–73). Clasping apparatus somewhat asymmetrical. Uncus stem narrow, short, dilated distally, with fine tip; scaphium membranous with weakly-sclerotized plate on subscaphium; valva elongated, narrowed at base, with acute apex; ampulla long, spine-like, curved towards costa, finely pointed, reaching apex of valva and costal margin. Aedeagus short, straight, with heavily sclerotized convex field on carina. Vesica globular, everted forward and recurved laterally; medial part membranous; subbasal diverticulum with small, heavily-sclerotized crown-like cornutus on top; 1^st^ medial diverticulum elliptical; 2^nd^ medial diverticulum large with crown-like cornutus on the top; ^1st^ terminal diverticulum two-chambered, scobinated, one of them elongated-tapering, another globular; 2^nd^ terminal diverticulum tapered; opening point of terminal tube located at base of medial part of vesica near carina, terminal tube membranous with narrow sclerotized crest at base and weak scobination near gonopore (starting point of ductus ejaculatorius). **Female genitalia** (Fig. 84). Ovipositor short, papillae anales small, hairy with long setae on apical edges. Apophyses anteriores slender with fine tip, apophyses posteriores thin, somewhat longer than apophyses anteriores. Antrum triangular, very narrow anteriorly, wide posteriorly, with straight lateral margins, ductus bursae absent. Corpus bursae membranous, ovoid.

##### Distribution.

North and western Iran.

#### Lygephila moellendorffi

(Herz, 1904)

http://species-id.net/wiki/Lygephila_moellendorffi

Fig. 8

##### Material examined.

Paralectotype, ♂ [North] Korea (ZISP).

##### Note.

The name of this taxon was erroneously written as *moellendorfii* in Poole (1989) and as *moellendorfi* in Kononenko et al. (1998) and Kononenko and Han (2007). The correct spelling of the species described by Herz in honour of Paul von Moellendorff as per original description was *moellendorffi*.

##### Diagnosis.

*Lygephila moellendorffi* is known only from two males representing the type series. The photo of the paralectotype was illustrated in Kononenko et al. (1998); the genitalia of the paralectotype was first illustrated by Kononenko and Han (2007). Surprisingly, this species is confusingly similar to *Lygephila subpicata*, displaying no differential features comparing the habitus and the genitalia structures of the two species. The similarly elongated forewings with pointed tips have the same pattern, especially the triangular reniform stigma with sharp extension on the inner corner and satellite streak-like spots are practically identical in the two taxa. The common features of the male genitalia are the similar shape of uncus and valvae with the similarly sized and shaped ampulla being also located subapically and reaching the apical valval margins. Both species have short and relatively wide aedeagus and vesica with characteristic subbasal and 2^nd^ medial diverticula topped by crown-like cornuti; the terminal diverticula are also similar. This striking resemblance suggests that they represent the same species, but the great distance between their ranges does not support this conclusion.

**Male genitalia** (Figs 33, 34). Clasping apparatus somewhat asymmetrical (left valva slightly wider). Uncus stem narrow, dilated distally, with fine tip; valva elongated, narrowed at base, with acute apex; ampulla long, spine-like, curved towards costa, finely pointed, reaching apex of valva and costal margin. Aedeagus short, straight, with heavily sclerotized convex field on carina. Vesica globular, everted forward and recurved laterally; medial part membranous; subbasal diverticulum with small, crown-like cornutus on top; 1^st^ medial diverticulum elliptical; 2^nd^ medial diverticulum large with crown-like cornutus on the top; 1^st^ terminal diverticulum located near base of 2^nd^ medial diverticulum. Female unknown.

##### Distribution.

North Korea.

#### Lygephila alaica

Remm, 1983

http://species-id.net/wiki/Lygephila_alaica

Figs 13 , 14

##### Material examined.

1 ♂ Tajikistan, Gissar Mts, distr. Varzob, vill. Kondara, 1150–1200 m, 17–18.VI.2012, leg. E. Rutjan, coll. O. Pekarsky, slide No: OP1819m; 1 ♀, Tajikistan, Khatlonskaya reg., Muminabadsky distr., Lidzhak, 2000 m, 27.V.2006, leg. O. Pak, coll. O. Pekarsky, slide No: OP1568f; 1 ♀, Uzbekistan, Hissarskiy range, Metchetli Mts, Shargunsay, 38°36'N, 67°57'E, 1550m, 30.May, 2004, leg. Z. Weidenhoffer, coll. M. Dvořák, slide No: OP1792f.

##### Diagnosis.

*Lygephila alaica* should be attributed to the *Lygephila lusoria* species-group on the basis of both the external and genital diagnostic characters. The elongated forewing with pointed apex is similar to those of all species of this species-group, particularly to eastern representatives, *Lygephila colorata* and *Lygephila subpicata*. The spine-like ampulla with long skewed base, the short and wide aedeagus, the characteristic vesica structure, especially the presence of the well-developed subbasal diverticulum with large cornutus in the male genitalia and the heavily sclerotized, funnel-shaped antrum with strongly asymmetrical ostium bursae in the female genitalia indicate the close relationship with the *Lygephila lusoria* species-group.

**Male genitalia** (Figs 29, 74, 75). Slightly asymmetrical (right valva somewhat narrower). Uncus stem narrow, moderately dilated distally, with fine tip; scaphium membranous with sclerotized plate on subscaphium; valva wide, elongated with almost parallel margins, apex rounded; ampulla dentiform with large plate-like lateral extension. Aedeagus straight, carina slightly dilated with transverse wrinkles. Vesica globular, everted laterally; medial part membranous; small subbasal diverticulum topped by heavily sclerotized plate-like cornutus with two teeth; 1^st^ and 2^nd^ medial diverticula small; 1^st^ distal diverticulum resembles high-heeled shoe, 2^nd^, 3^rd^ and 4^th^ distal diverticula roughly equal in size and similar in shape; opening point of terminal tube located at base of medial part of vesica near carina, membranous with weak scobination from middle towards gonopore. **Female genitalia** (Fig. 85). Ovipositor relatively short, broad, papillae anales hairy with short setae on apical edges. Apophyses anteriores slender, apophyses posteriores thin, more than two times longer than apophyses anteriores. Antrum wide, short, ostium bursae asymmetrical, posterior margin with skewed concavity; ductus bursae as large as antrum, heavily sclerotized. Appendix bursae small, corpus bursae membranous, ovoid.

##### Distribution.

Central Asia – Tajikistan and Uzbekistan.

**Figures 39, 40.**
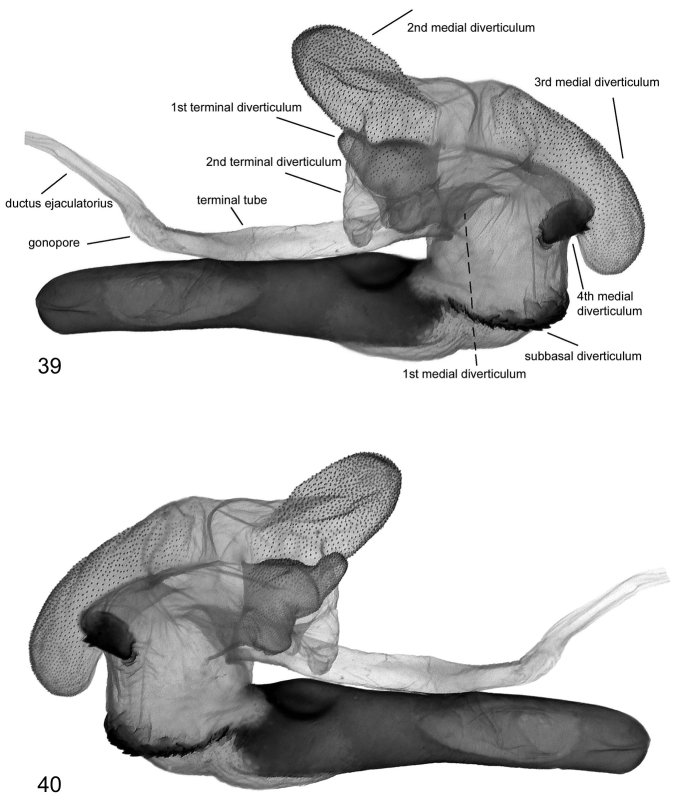
Vesica structure of *Lygephila lusoria lusoria* Hungary, slide No. OP1953m **39** dorsal view **40** ventral view.

**Figures 41, 42.**
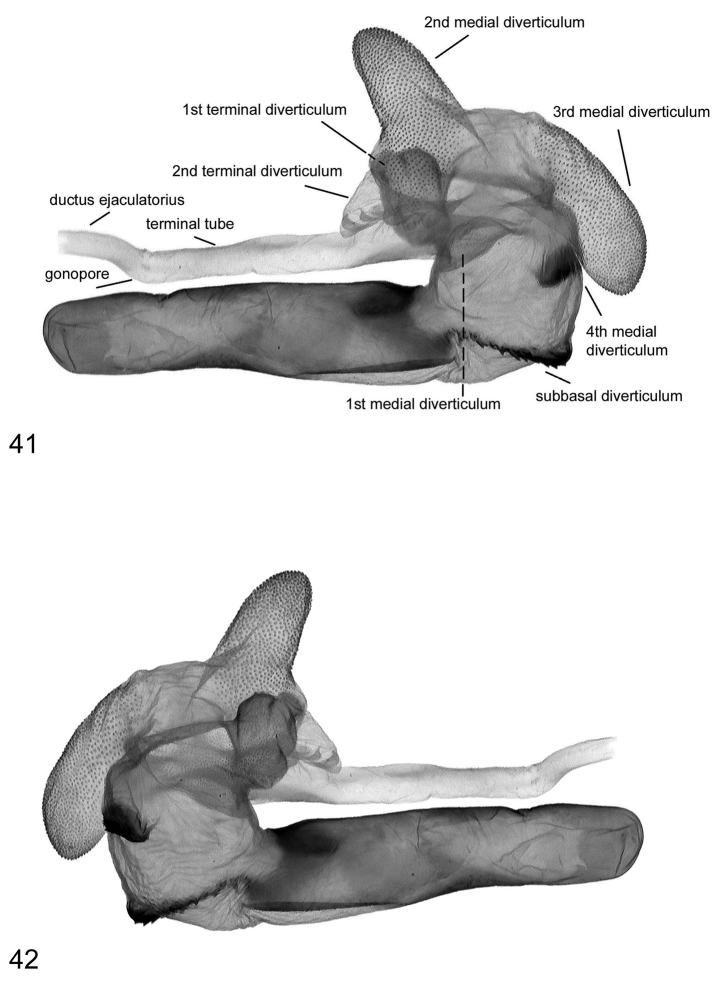
Vesica structure of *Lygephila lusoria glycyrrhizae* Spain, slide No. OP1977m **41** dorsal view **42** ventral view.

**Figures 43–46.**
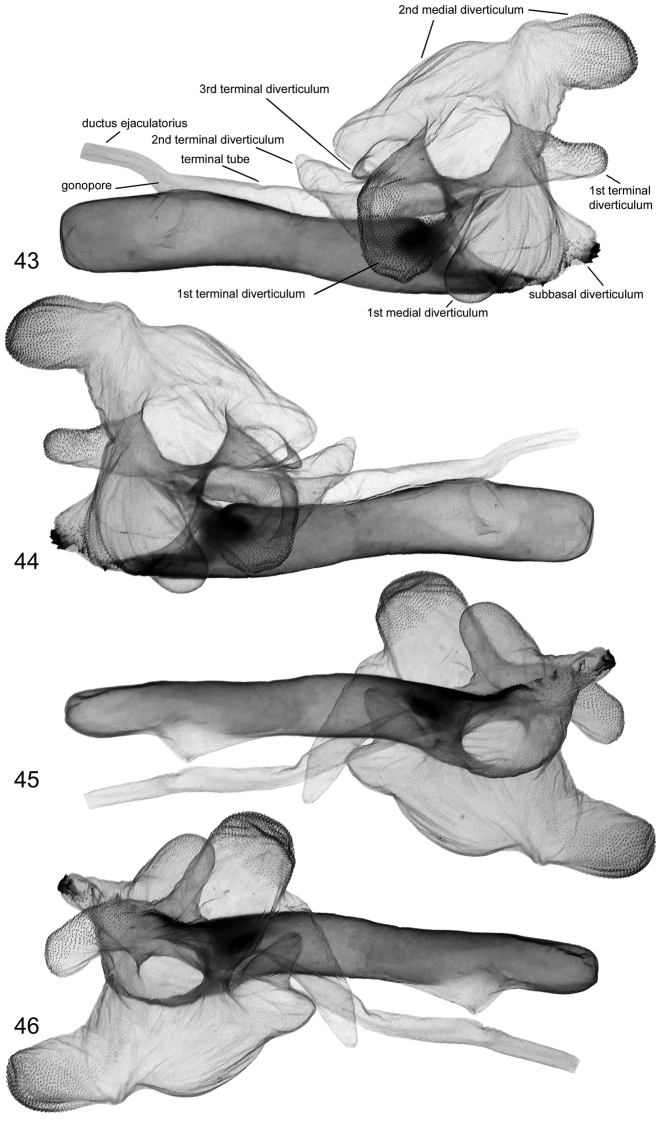
Vesica structure of *Lygephila amasina* Turkey, slide No. OP2260m **43** dorsal view **44** ventral view **45** lateral view **46** lateral view opposite side.

**Figures 47–49.**
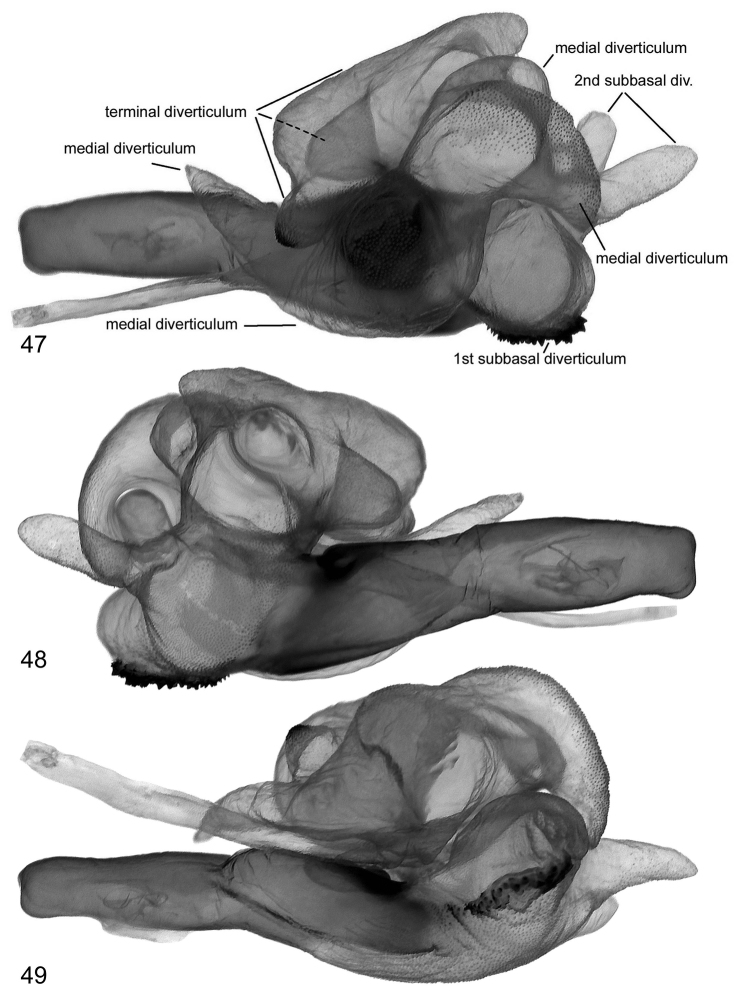
Vesica structure of *Lygephila colorata* paratype, Pakistan, slide No. OP1969m **47** dorsal view **48** ventral view **49** sublateral view.

**Figures 50, 51.**
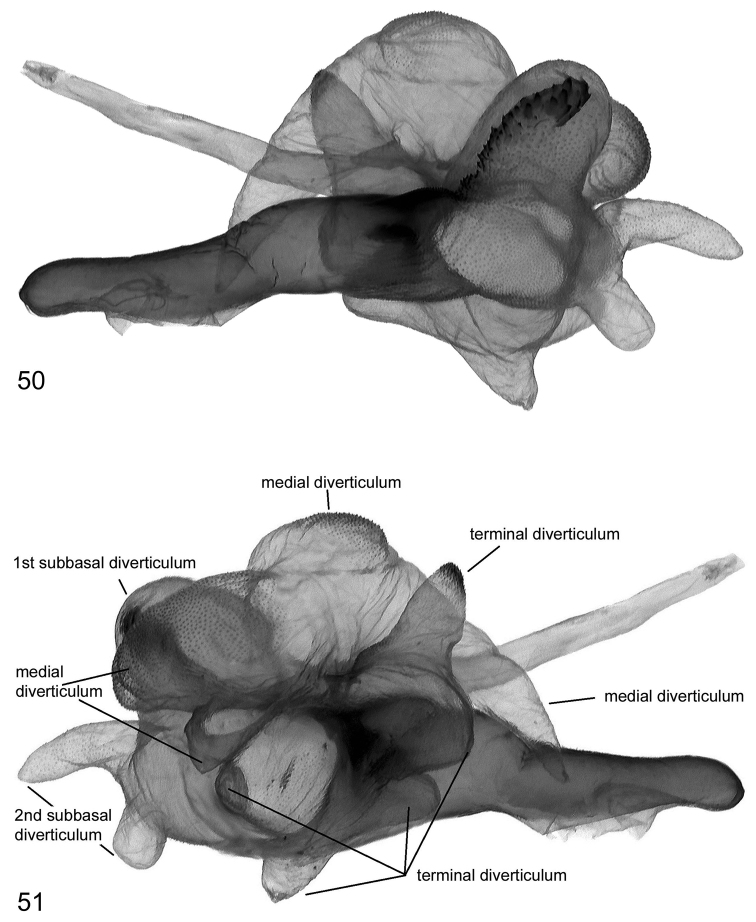
Vesica structure of *Lygephila colorata* paratype, Pakistan, slide No. OP1969m **50** lateral view **51** lateral view opposite side.

**Figures 52–54.**
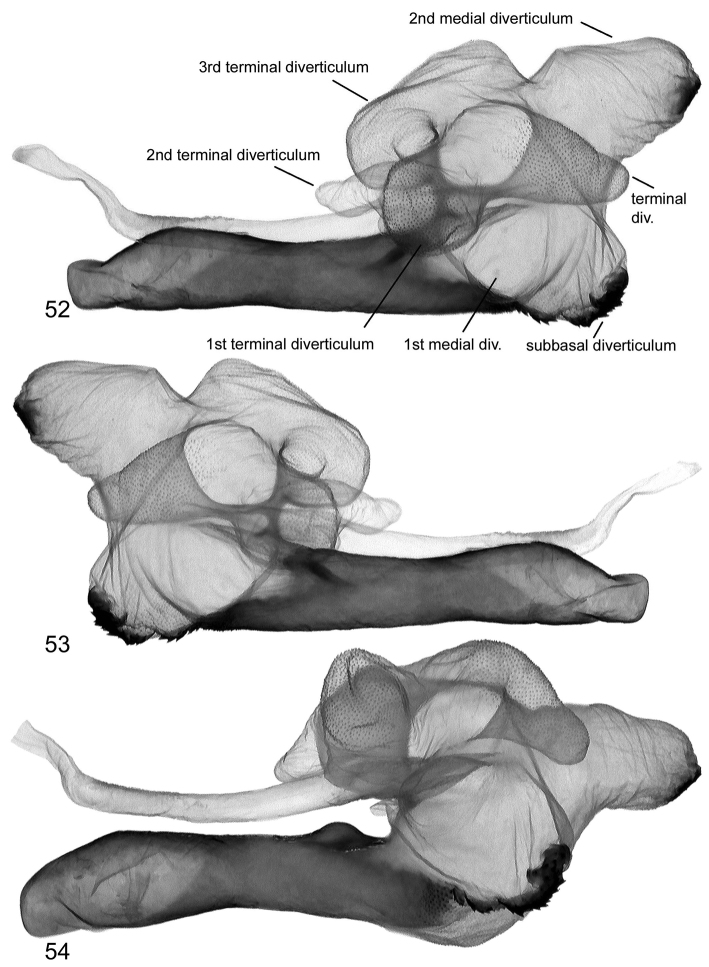
Vesica structure of *Lygephila pallida* Cotype, Turkey, Zeitun, slide No. OP1933m **52** dorsal view **53** ventral view **54** subdorsal view.

**Figures 55–57.**
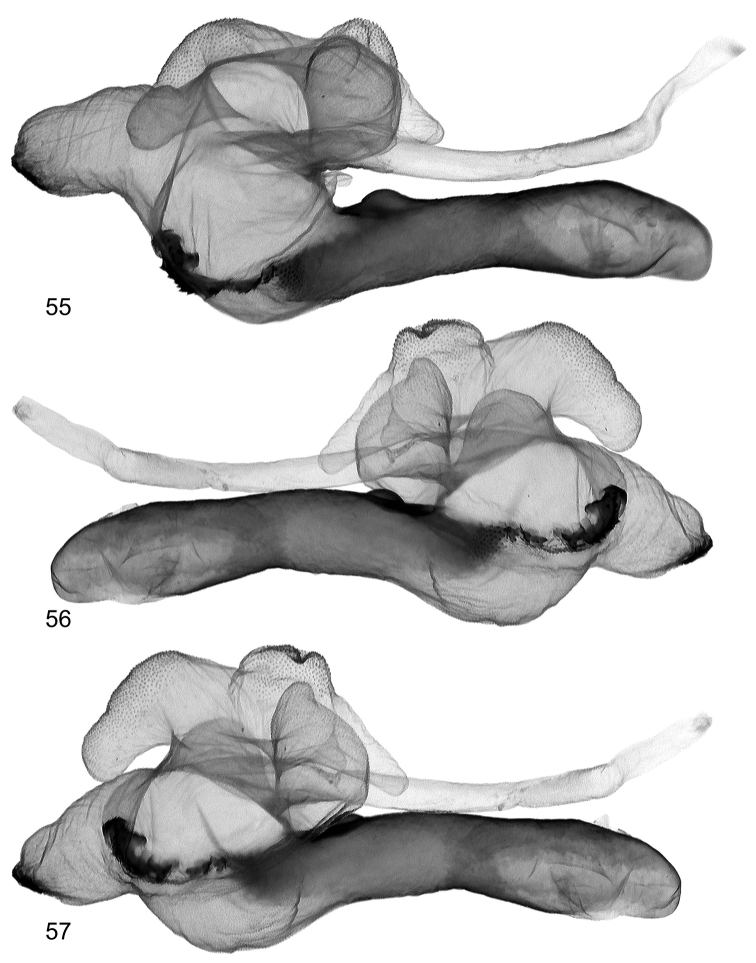
Vesica structure of *Lygephila pallida* Cotype, Turkey, Zeitun, slide No. OP1933m **55** subdorsal view opposite side **56** sublateral view **57** sublateral view opposite side.

**Figures 58, 59.**
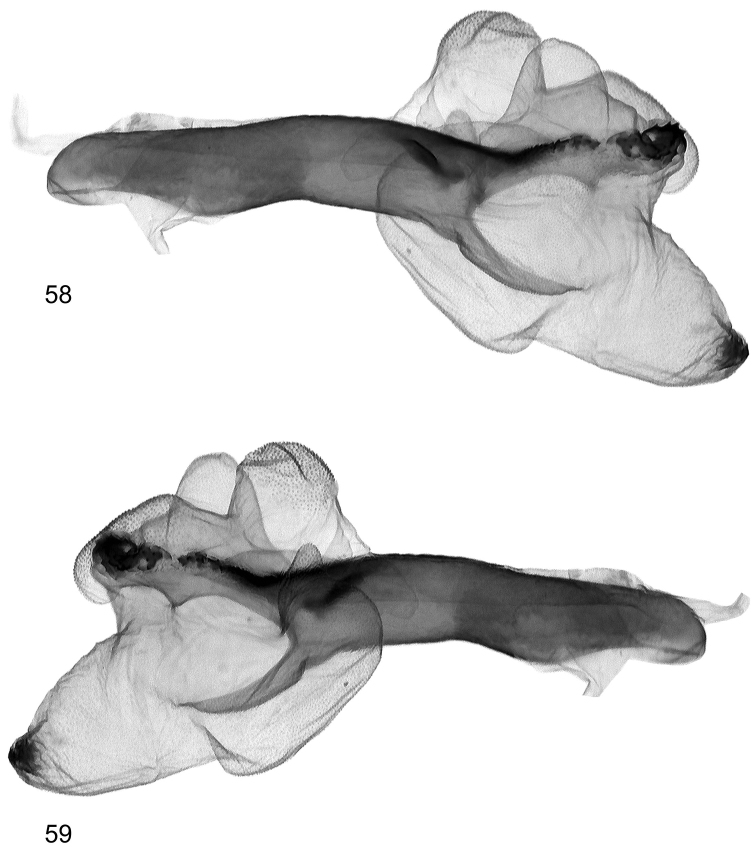
Vesica structure of *Lygephila pallida* Cotype, Turkey, Zeitun, slide No. OP1933m **58** lateral view **59** lateral view opposite side.

**Figures 60–62.**
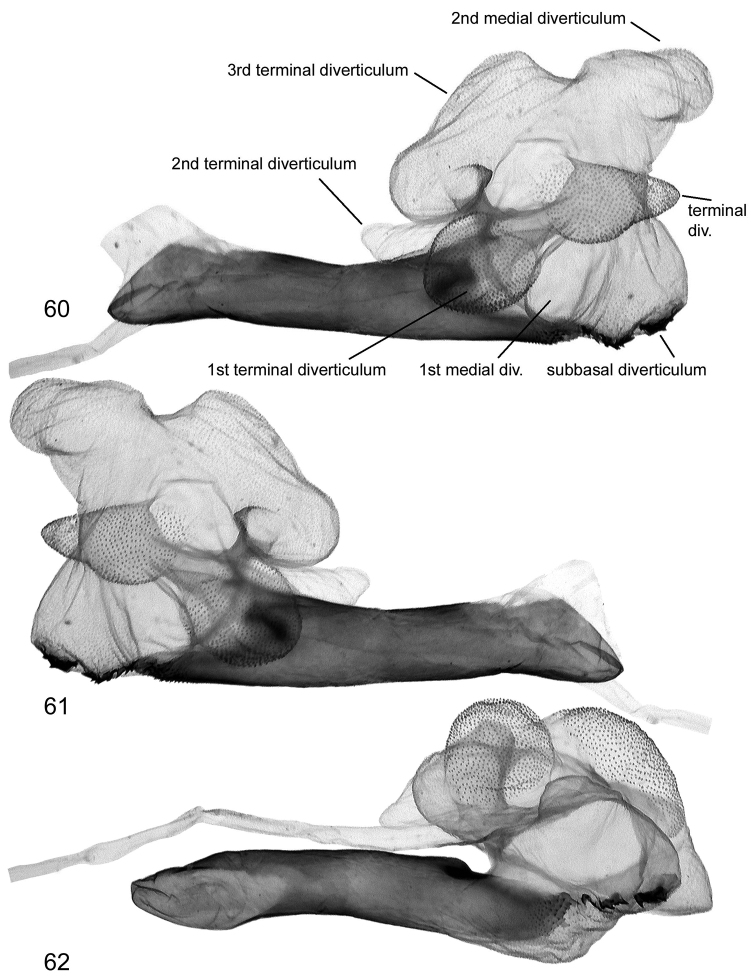
Vesica structure of *Lygephila minima* sp. n. paratype, South Russia, Stavropol krai, slide No. OP1607m **60** dorsal view **61** ventral view **62** subdorsal view.

**Figures 63–65.**
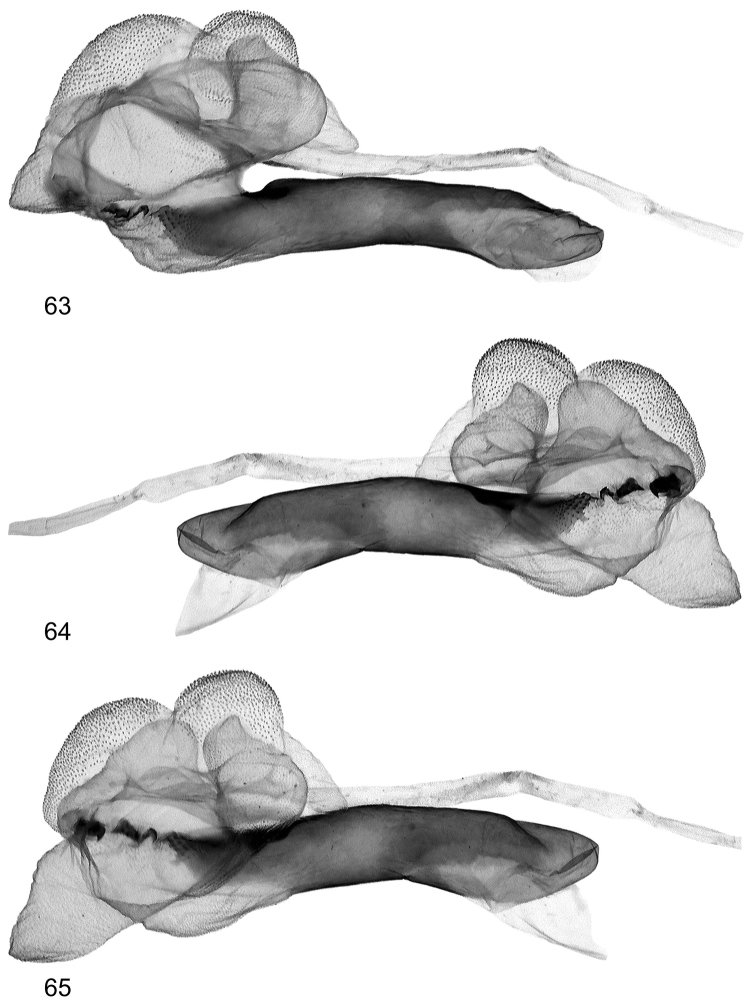
Vesica structure of *Lygephila minima* sp. n. paratype, South Russia, Stavropol krai, slide No. OP1607m **63** subdorsal view opposite side **64** sublateral view **65** sublateral view opposite side.

**Figures 66, 67.**
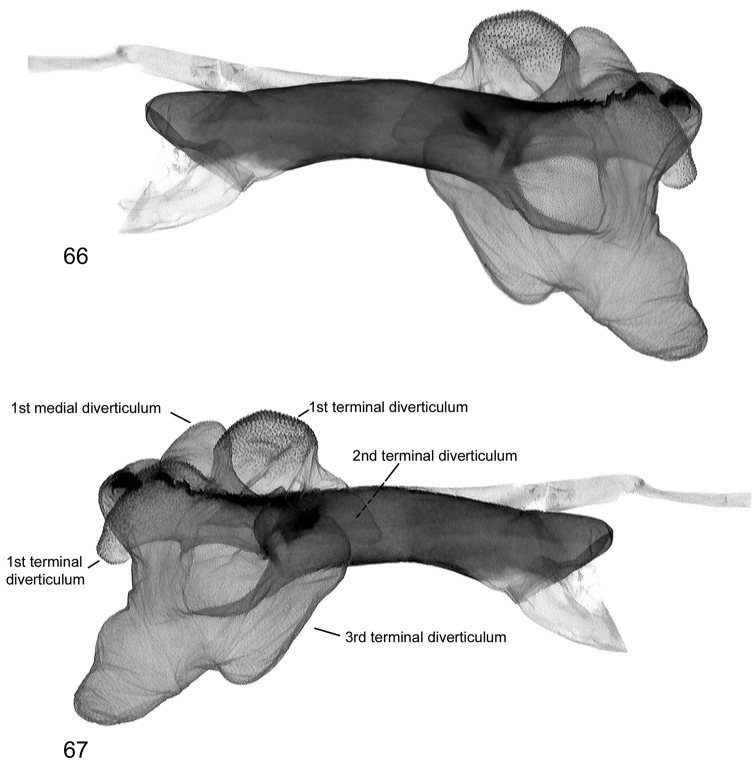
Vesica structure of *Lygephila minima* sp. n. paratype, South Russia, Stavropol krai, slide No. OP1607m **66** lateral view **67** lateral view opposite side.

**Figures 68–70.**
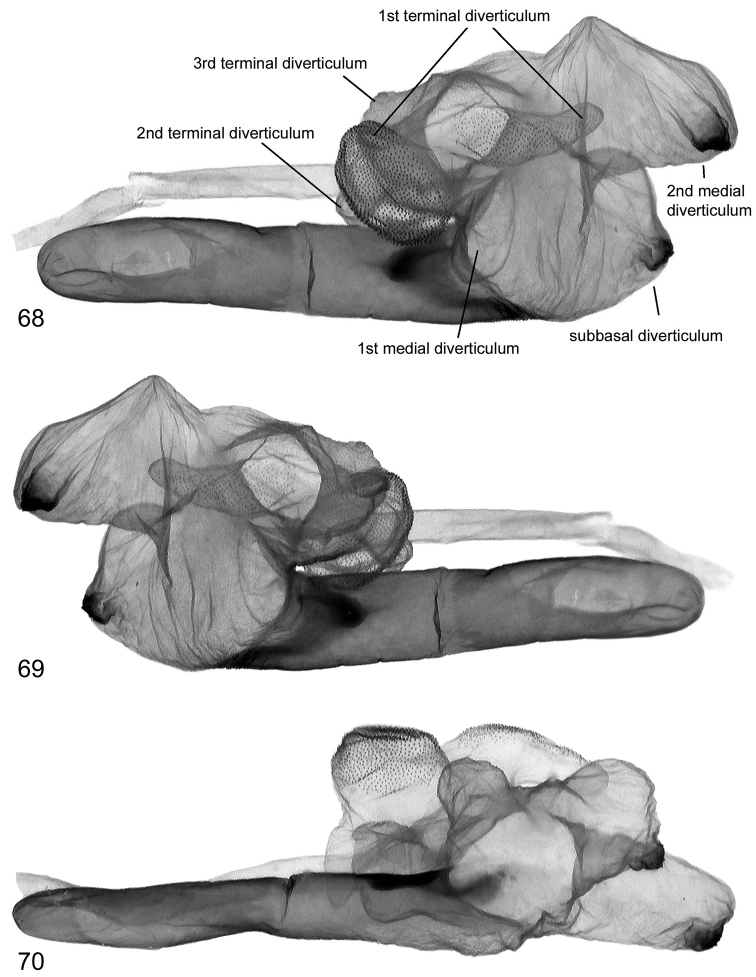
Vesica structure of *Lygephila subpicata* Iran, Prov. Fars, slide No. OP2002m **68** dorsal view **69** ventral view **70** sublateral view.

**Figures 71–73.**
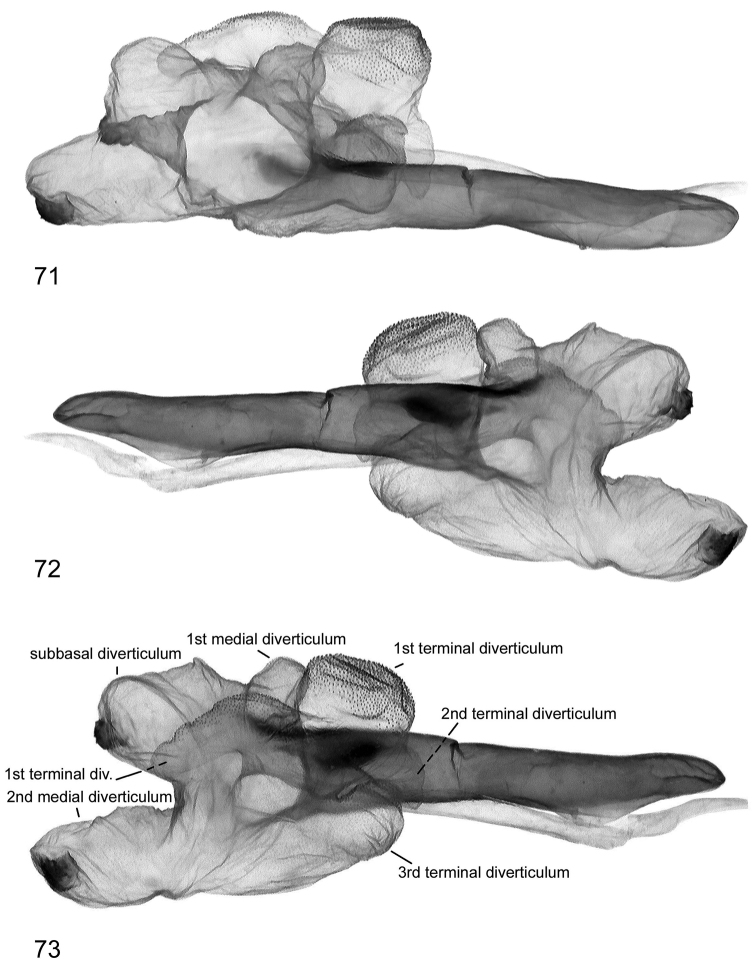
Vesica structure of *Lygephila subpicata* Iran, Prov. Fars, slide No. OP2002m **71** sublateral view opposite side **72** lateral view **73** lateral view opposite side.

**Figures 74, 75.**
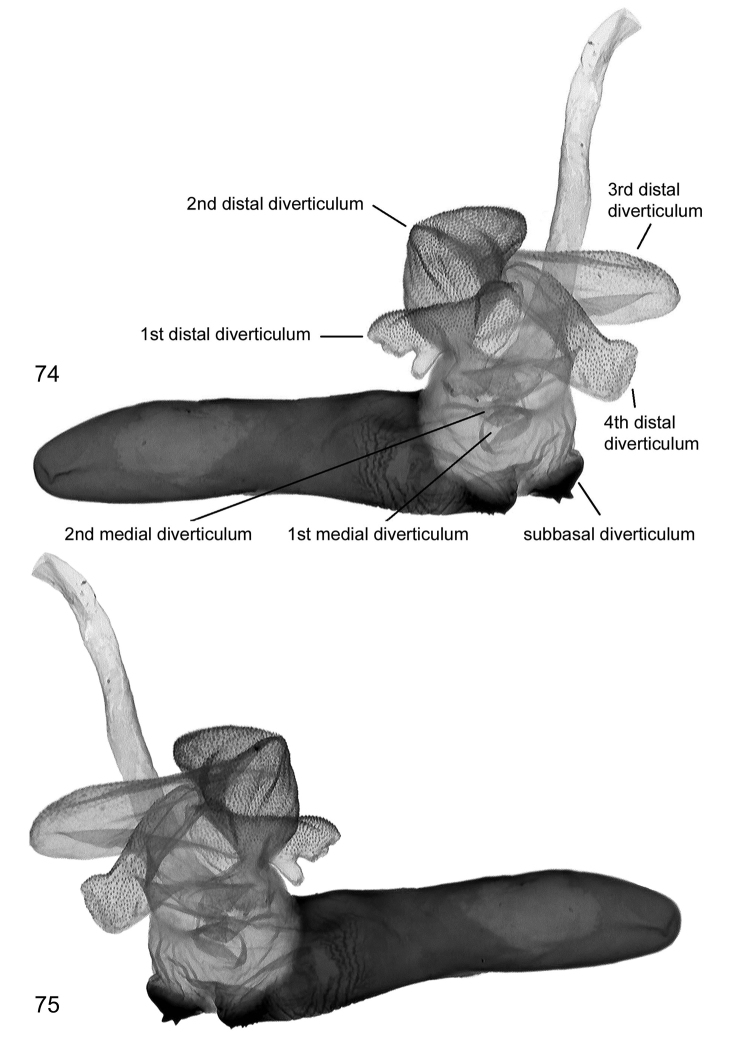
Vesica structure of *Lygephila alaica* Tajikistan, Gissar Mts, slide No. OP1819m **74** dorsal view **75** ventral view.

**Figures 76–85.**
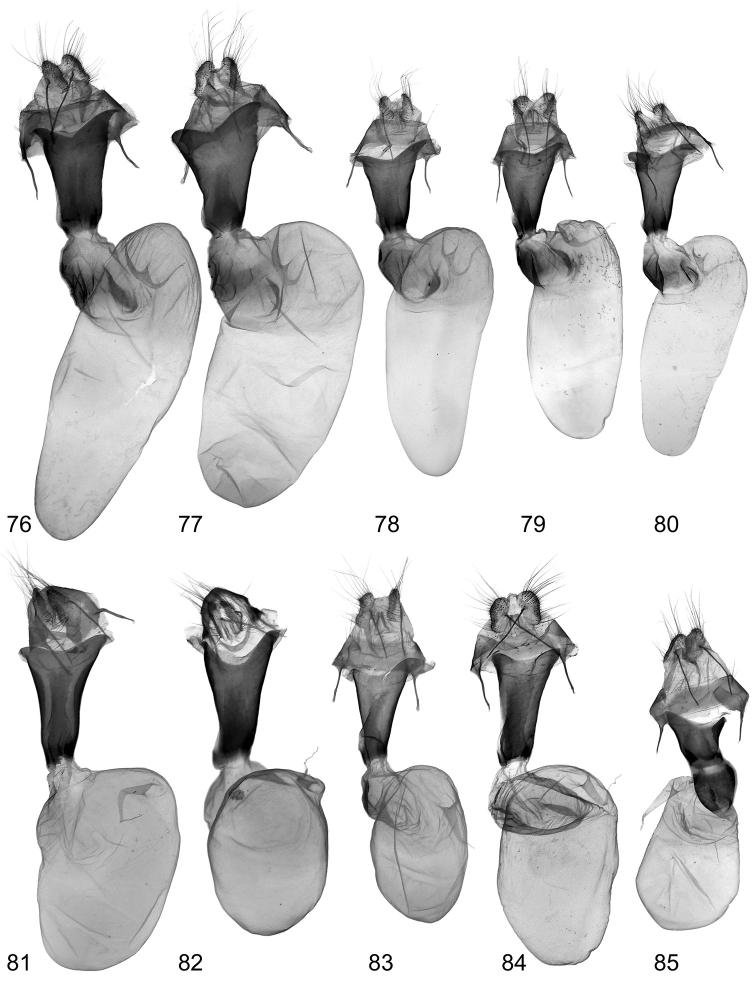
Female genitalia. 76, **77** *Lygephila lusoria lusoria* **76** Hungary, slide No. OP1954f **77** Ukraine, Crimea, slide No. OP2053f **78–80** *Lygephila lusoria glycyrrhizae* **78** Spain, slide No. OP1978f **79** Spain, slide OP2137f **80** Spain, slide No. OP2265f **81** *Lygephila amasina* Lebanon, slide No. OP1960f **82** *Lygephila colorata* paratype, Pakistan, slide No. OP1970f **83** *Lygephila pallida* Turkey, Palandoeken, slide No. OP2070f **84** *Lygephila subpicata* paratype, Iran, Semnan, slide No. OP2060f **85** *Lygephila alaica* Tajikistan, Gissar Mts, slide No. OP1568f.

## Supplementary Material

XML Treatment for Lygephila lusoria lusoria

XML Treatment for Lygephila lusoria glycyrrhizae

XML Treatment for Lygephila amasina

XML Treatment for Lygephila colorata

XML Treatment for Lygephila pallida

XML Treatment for Lygephila fereidun

XML Treatment for Lygephila minima

XML Treatment for Lygephila subpicata

XML Treatment for Lygephila moellendorffi

XML Treatment for Lygephila alaica
